# SGDriver: a novel structural genomics-based approach to prioritize cancer related and potentially druggable somatic mutations

**DOI:** 10.1186/1471-2105-16-S15-P21

**Published:** 2015-10-23

**Authors:** Junfei Zhao, Feixiong Cheng, Zhongming Zhao

**Affiliations:** 1 Department of Biomedical Informatics, Vanderbilt University School of Medicine, Nashville, TN 37203, USA; 2 Department of Cancer Biology, Vanderbilt University School of Medicine, Nashville, TN 37232, USA; 3 Department of Psychiatry, Vanderbilt University School of Medicine, Nashville, TN 37232, USA

## Background

A huge volume of somatic mutations have been generated through large cancer genome sequencing projects such as The Cancer Genome Atlas (TCGA) and the International Cancer Genome Consortium (ICGC). However, understanding the functional consequences of somatic mutations in cancer and translating the results into clinical use remains a major challenge in cancer genomic studies. Thanks to the rapid development of structural genomic technologies, such as X-ray and NMR, large amounts of protein structure data have been generated during the past decade, which enables us to map somatic mutations to protein functional features (i.e., protein-ligand binding sites) and investigate their potential impacts[1,2].

## Materials and methods

In this study, we developed SGDriver, a structural genomics-based approach that incorporates protein-ligand binding sites information into the somatic missense mutation data to help understand the pathophysiological role of variations and prioritize putative druggable mutations using a Bayes inference statistical framework. We applied SGDriver to 746,631 missense mutations across 16 major cancer types from TCGA.

## Results

We found 251 genes enriched with ligand binding site mutations in their protein products with false discovery rate less than 0.05, including 43 Cancer Gene Census (CGC) genes. Furthermore, drug-gene network analysis identifies ~100 druggable anticancer targets using the data from DrugBank, Therapeutics Target Database, and PharmGKB databases. Finally, bioinformatics analysis using Connectivity Map data identified several existing drugs that may be potentially repurposed for precision cancer therapy by targeting cancer driver gene products identified by our SGDriver method. In summary, this study provides a novel computational approach to identify new druggable mutations for precision cancer medicine.
